# *Caenorhabditis elegans* expressing the *Saccharomyces cerevisiae* NADH alternative dehydrogenase Ndi1p, as a tool to identify new genes involved in complex I related diseases

**DOI:** 10.3389/fgene.2015.00206

**Published:** 2015-06-11

**Authors:** Raynald Cossard, Michela Esposito, Carole H. Sellem, Laras Pitayu, Christelle Vasnier, Agnès Delahodde, Emmanuel P. Dassa

**Affiliations:** I2BC, Institute for Integrative Biology of the Cell, CEA, CNRS, Université Paris-SudOrsay, France

**Keywords:** complex I, *Caenorhabditis elegans*, RNAi screening, Ndi1p, embryonic lethality

## Abstract

Isolated complex I deficiencies are one of the most commonly observed biochemical features in patients suffering from mitochondrial disorders. In the majority of these clinical cases the molecular bases of the diseases remain unknown suggesting the involvement of unidentified factors that are critical for complex I function. The *Saccharomyces cerevisiae NDI1* gene, encoding the mitochondrial internal NADH dehydrogenase was previously shown to complement a complex I deficient strain in *Caenorhabditis elegans* with notable improvements in reproduction and whole organism respiration. These features indicate that Ndi1p can functionally integrate the respiratory chain, allowing complex I deficiency complementation. Taking into account the Ndi1p ability to bypass complex I, we evaluate the possibility to extend the range of defects/mutations causing complex I deficiencies that can be alleviated by *NDI1* expression. We report here that *NDI1* expressing animals unexpectedly exhibit a slightly shortened lifespan, a reduction in the progeny, and a depletion of the mitochondrial genome. However, Ndi1p is expressed and targeted to the mitochondria as a functional protein that confers rotenone resistance to those animals without affecting their respiration rate and ATP content. We show that the severe embryonic lethality level caused by the RNAi knockdowns of complex I structural subunit encoding genes (e.g., NDUFV1, NDUFS1, NDUFS6, NDUFS8, or GRIM-19 human orthologs) in wild type animals is significantly reduced in the Ndi1p expressing worm. All together these results open up the perspective to identify new genes involved in complex I function, assembly, or regulation by screening an RNAi library of genes leading to embryonic lethality that should be rescued by *NDI1* expression.

## Introduction

In humans, NADH: ubiquinone oxidoreductase (complex I) is composed of at least 45 different subunits encoded by both mitochondrial and nuclear genomes making it the largest of the five respiratory chain complexes. It catalyzes electron transfer from NADH to the respiratory chain through ubiquinone as electron acceptor. This huge complex is composed of structural subunits organized in three well-defined functional modules: the N module involved in oxidizing NADH, the Q module involved in reducing ubiquinone and the P module dedicated to the proton translocation (Brandt, 2006). Furthermore, the proper sequential assembly of this huge complex requires many non-structural assembly factors and this process remains partially a conundrum (Pagniez-Mammeri et al., 2012b).

Mitochondrial dysfunctions caused by respiratory chain complex I deficiency have been shown to possibly originate from deleterious mutations in either the nuclear or the mitochondrial genomes, resulting in a wide spectrum of human diseases ranging from leber hereditary optic neuropathy (LHON), that specifically targets one organ, to diseases affecting several organs as the mitochondrial encephalomyopathy associated with cardiomyopathy (Fassone and Rahman, 2012). Isolated complex I deficiency is encountered in 23 to 32% of the mitochondrial diseases, which makes it the most frequent biochemical signature found in these disorders (Scaglia et al., 2004; Hoefs et al., 2012). Given the important number of either mitochondrial or nuclear genes that can be mutated, the molecular diagnosis of these disorders is still a hard task to perform. A so challenging task that only around 20% of the patients harboring a complex I deficiency carry a mutation in one of the known complex I subunit encoding genes (Rotig, 2010). This observation suggests the existence of many uncharacterized genes implicated in complex I function, assembly or regulation. A recent exome sequencing screening, for mutations in 75 complex I associated genes in about 150 patients with a biochemically defined complex I deficiency identified mutations in 50% of the cases and causative mutations in less than 20% of the patients (Haack et al., 2012).

To overcome this difficulty we propose to develop a genomic large-scale RNAi screening in the worm *Caenorhabditis elegans* to identify new complex I related genes by taking advantage of a transgenic worm expressing the yeast NADH alternative dehydrogenase (Ndi1p). Indeed, in contrast to mammals, the respiratory chain in fungi and plants is composed of multiple dehydrogenases that together allow a precise regulation of the electrons channeling in the respiratory chain, thus providing bypasses of complex I (Rasmusson et al., 2008). In the yeast *Saccharomyces cerevisiae* complex I is merely replaced by a set of three monomeric non-proton pumping NADH dehydrogenases. Indeed, the internal mitochondrial membrane contains two NADH dehydrogenases facing the inner membrane space (Nde1p and Nde2p) and one facing the matrix (Ndi1p) (Marres et al., 1991; Luttik et al., 1998). *S. cerevisiae* Ndi1p was shown to be enzymatically active when expressed in mammals (Yagi et al., 2006; Marella et al., 2008, 2010; Cannino et al., 2012), *Drosophila* (Sanz et al., 2010), and nematode (DeCorby et al., 2007). The complex I bypass established by Ndi1p was demonstrated to be well tolerated. It protects rat neurons against the specific complex I inhibitor, rotenone, and rescues complex I deficiency in several organisms *in vivo* and *in vitro* (DeCorby et al., 2007; Marella et al., 2008, 2010; Maas et al., 2010; Sanz et al., 2010; Cho et al., 2012; Chadderton et al., 2013). In the nematode *C. elegans*, Lemire and coworkers reported that Ndi1p expression compensates the phenotypes of a point mutation in *nuo-1* (NDUFV1 worm ortholog, encoding a catalytic complex I subunit). This improvement is associated with the restoration of the membrane potential although *NDI1* is carried by an extrachromosomal array and the worm is a mosaic animal (DeCorby et al., 2007).

*Caenorhabditis elegans* presents a fully sequenced genome that encodes proteins among which 83% have an human ortholog (Lai et al., 2000). The respiratory chain is well conserved between worms and humans, with five canonical complexes. Furthermore, their composing subunits are characterized by a high degree of sequence similarity (Tsang and Lemire, 2003; Rea et al., 2010). For example, 38 complex I subunits (7 and 31 from mitochondrial and nuclear genomes, respectively) are conserved (Falk et al., 2009). In *C. elegans*, mitochondrial dysfunctions are known to cause developmental arrest or retardation at either embryonic or larval stage as well as longevity alteration (Kamath et al., 2003; Lee et al., 2003; Tsang and Lemire, 2003; Dancy et al., 2014). In a genomic large-scale screening, the phenotypes observed for 21 known structural complex I genes silenced by RNAi are embryonic lethal (81%), larval stage arrest (5%), and developmental delay (14%) (Kamath et al., 2003). The beneficial effect of Ndi1p expression in the worm was reported for a transgenic complex I mutant (*nuo-1* A352V, corresponding to A341 in human NDUFV1; DeCorby et al., 2007) but not for complex I subunit RNAi knockdown in wild type animals yet.

In the present work we evaluated and demonstrated the feasibility of an original strategy to identify unknown complex I related genes based on the complementation capability offered by the bypassing effect of the yeast Ndi1p when expressed in *C. elegans*.

We first characterized the consequences of *NDI1* expression in wild type worms and found that *NDI1* expression is not as benign as observed in other organisms (Sanz et al., 2010). *NDI1* expression leads to a decrease in brood size, longevity, and mtDNA content without any detectable effect on the respiration rate. However, the enzyme is well active, increasing rotenone resistance of animal.

Despite these phenotypes, we demonstrated that Ndi1p was able to overcome reduced brood size and embryonic lethality due to RNAi knockdown of several known complex I genes. This efficient Ndi1p bypass now allows us to develop a new approach aiming to identify in *C. elegans* new genes involved in complex I function.

## Materials and Methods

### Worm Strains and Maintenance

We used the following two *C. elegans* strains: N2 (Bristol) wild type; N2-NDI1 (LB56), uaEx38 (plet-858::NDI1, pTG96, pPD118.25NEO; kindly provided by Bernard Lemire), which carries the *plet-858::NDI1* expression plasmid, the pTG96 plasmid expressing a sur-5::GFP fusion protein that localizes to nuclei and the pPD118.25NEO plasmid conferring the G418 resistance.

Worms were cultured at 20°C on nematode growth medium (NGM) plates seeded with OP50 *Escherichia coli* strain and supplemented with 1 mg/mL of G418 antibiotic to select for the retention of the extrachromosomal arrays of the N2-NDI1 line. When G418 is not used adult worms have to be sorted out for the presence of *NDI1* transgene by their GFP staining.

### Worm Phenotyping

To measure brood size, three synchronized L4 animals were transferred on plates seeded with OP50 bacteria until the adult stage. Plates were monitored each day and animals transferred to new plates. Laid eggs were scored each day and plates were incubated one more day at 20°C to score hatching. This was done until the end of the egg laying period.

To analyze the development of larvae from L1–L4, freshly hatched L1 animals were incubated at 20°C, monitored every day and scored until the L4 stage.

For lifespan analysis, 100 synchronized L4 larvae were transferred on NGM plates seeded with OP50 bacteria. Adults were counted each day and scored as dead if they did not respond to gentle stimulation. Adults were transferred daily to new plates during the egg laying period. Survival curves were further analyzed using the log-rank test.

Rotenone-protection assay was performed as described in (Saha et al., 2009).

### Isolation of Mitochondria

Adult worms grown on NGM plates supplemented with G418 in the case of N2-NDI1 line were collected, rinsed three times in M9 medium, concentrated by centrifugation and frozen. Isolation of mitochondria was performed as described in (van den Ecker et al., 2010) excepted that we used glass beads in a FastPrep (MP Biomedical) for the release of the mitochondria.

### Western Blot Analysis

Isolated mitochondria from young synchronized adult worms were used to perform Western blot analysis. The rabbit polyclonal antibody directed against *S. cerevisiae* Ndi1p was used at 1/10,000 and mouse monoclonal antibody directed against human NDUFS3 (Mitosciences, MS112) was used at 1/1,000. Secondary antibodies used were, respectively, HRP conjugated goat anti-rabbit and HRP conjugated sheep anti-mouse (GE healthcare) at 1/10,000.

### Total Worm Respiration

Oxygen consumption rates were measured using an Oxytherm oxygen electrode (Hansatech, Norfolk, UK). Synchronized worms were grown on NGM seeded with OP50 bacteria or HT115 bacteria expressing the dsRNA of interest. In the case of N2-NDI1 worms, transgenic animals were selected upon G418 resistance. Synchronized worms were washed three times with M9 buffer before being introduced in the measurement chamber maintained at 20°C. Nematodes were counted using triplicate aliquots. At least around 1000 worms were used for each measurement to reach sufficient respiration rates.

### Ethidium Bromide Assay

Ethidium bromide (EtBr) assay was performed as described in (Addo et al., 2010). Young synchronized adult worms were transferred to fresh NGM plates with or without different concentrations of EtBr. Worms were allowed to lay at least 100 eggs before being removed. Eggs were immediately counted and the F1 progeny produced was analyzed after 3 and 4 days. At day 4, evaluation of the F1 progeny arrested at the L3 stage was compared to the number of adults on the same plate. Data are the mean ± SD of three independent experiments.

### Quantification of mtDNA

Total worm DNA extractions were performed using the Nucleopsin Tissue Extraction Kit (Macherey-Nagel) from 15 synchronized worms taken at day 8 of adulthood and no longer reproductive. The mitochondrial *cyt-b* and the nuclear T04C12.4 (*act-3*) genes were amplified independently by quantitative real-time qPCR using the following primers: cyt-bf/cyt-br TTCCAATTTGAGGGCCAACT/AACTAGAATAGCTCACGGCAATAAAA) and act-3f/act-3r (TGCGACATTGATATCCGTAAGG/GGTGGTTCCTCCGGAAAGAA). Amplifications were performed in triplicates using the Maxima SYBR Green Master Mix (Thermo scientific) in a LightCycler real-time qPCR system (Roche). Amplification efficiencies were determined for each couple of primers, based on standard curves established using serial dilutions of one of the DNA samples. Data were analyzed with software using the “second derivative” method of quantification.

Data represents the mean ratio ± SEM of mitochondria over nuclear gene copy number for three independent experiments. Significance was determined by student’s *t*-test.

### RNA Interference

The RNAi experiments were performed using the feeding procedure described (Kamath and Ahringer, 2003) with slight modifications. Feeding RNAi clones were purchased from the Ahringer RNAi library (Geneservice Limited) and sequenced. Exponential cultures of HT115 (DE3) bacteria (RNaseIII-deficient *E. coli* strain, with an IPTG-inducible T7-RNA polymerase) carrying the plasmid expressing the dsRNA of interest were seeded on NGM plates supplemented with 1 mM IPTG, 25 μg/mL carbenicillin, and incubated at 20°C for 48 h to allow the expression of the double-stranded RNA (dsRNA). Worms fed by HT115 bacteria carrying the L4440 vector were used as control in all experiments.

For L1 RNAi, synchronized L1-stage worms were placed onto RNAi plates and incubated 24 h at 20°C and for 72 h at 15°C. Then, three synchronized young adult worms were picked up and transferred to fresh RNAi plates. For N2-NDI1 worms, animals were selected according to GFP staining. For L3 RNAi, the same protocol was applied except that L1 worms were grown on NGM plates seeded with OP50 bacteria until the L3 stage. In the case of N2-NDI1 animals the medium also contains G418, which allows the selection of the NDI1 expressing worms. L3 were washed twice in M9 medium to eliminate most OP50 bacteria and then transferred on RNAi plates (without G418) and incubated 72 h at 15°C.

Adult worms were allowed to lay eggs on RNAi plates for 6 h before being transferred to another plate overnight. The day after adult worms were sacrificed, laid eggs were immediately counted and the F1 progeny produced was analyzed. Embryonic lethality level of the F1 progeny was quantified by counting at least 100 eggs laid and hatching was scored 24 h later. Egg laying and hatching were thus scored on a period corresponding to the maximum amount of eggs laid.

### RNA Extraction and Quantification

RNAs were extracted from 3 independent batches of 30 synchronized N2 and N2-NDI adults worms grown in L3 RNAi conditions (worms fed with HT115 bacteria expressing (+) dsRNA designed to knock down the expression of the CO9H10.3 and C34B2.8 genes. Trizol (InVitrogen) RNA extraction was followed with DNAse treatment using the DNA-free kit (Ambion) and Reverse transcription using the SuperScript Vilo cDNA synthesis Kit (InVitrogen). The abundance of CO9H10.3 and C34B2.8 transcripts were determined by real time qRT-PCR, respectively, using the couple of primers CAGTGTACTCCATGCCGTG/GGCGAATGAGTCCCTGAAC and TCCAACTGCTACTGAGGTATT/CCACTTGGACATCCATTGTG. The specific amplification of cDNA is attested by the absence of amplification products on identical samples in which the reverse transcriptase was omitted. The abundance of *ama-1* (F36A4.7) transcripts was used as a reference (Hoogewijs et al., 2008; Addo et al., 2010; van den Ecker et al., 2012) and determined using the couple of primers CGACATACAATCCAACATCTC/GTTGGAGAGTACTGAGCCG. In N2 and N2-NDI worms *ama-1* is expressed at identical level and in equivalent levels if compared to NDUFV1 and GRIM-19 transcripts.

### ATP Content Measurement

The total ATP content from 3 batches of 1000 L4 synchronized worms for each line was measured according to the procedure described in (Braeckman et al., 2002). Quantification was performed using the ATPlite bioluminescence Kit (PerkinElmer) and normalization was done by protein determination (Bradford, Biorad) on three equivalent batches of worms.

## Results

### Characterization of the N2-NDI1 Worm

**FIGURE 1:**
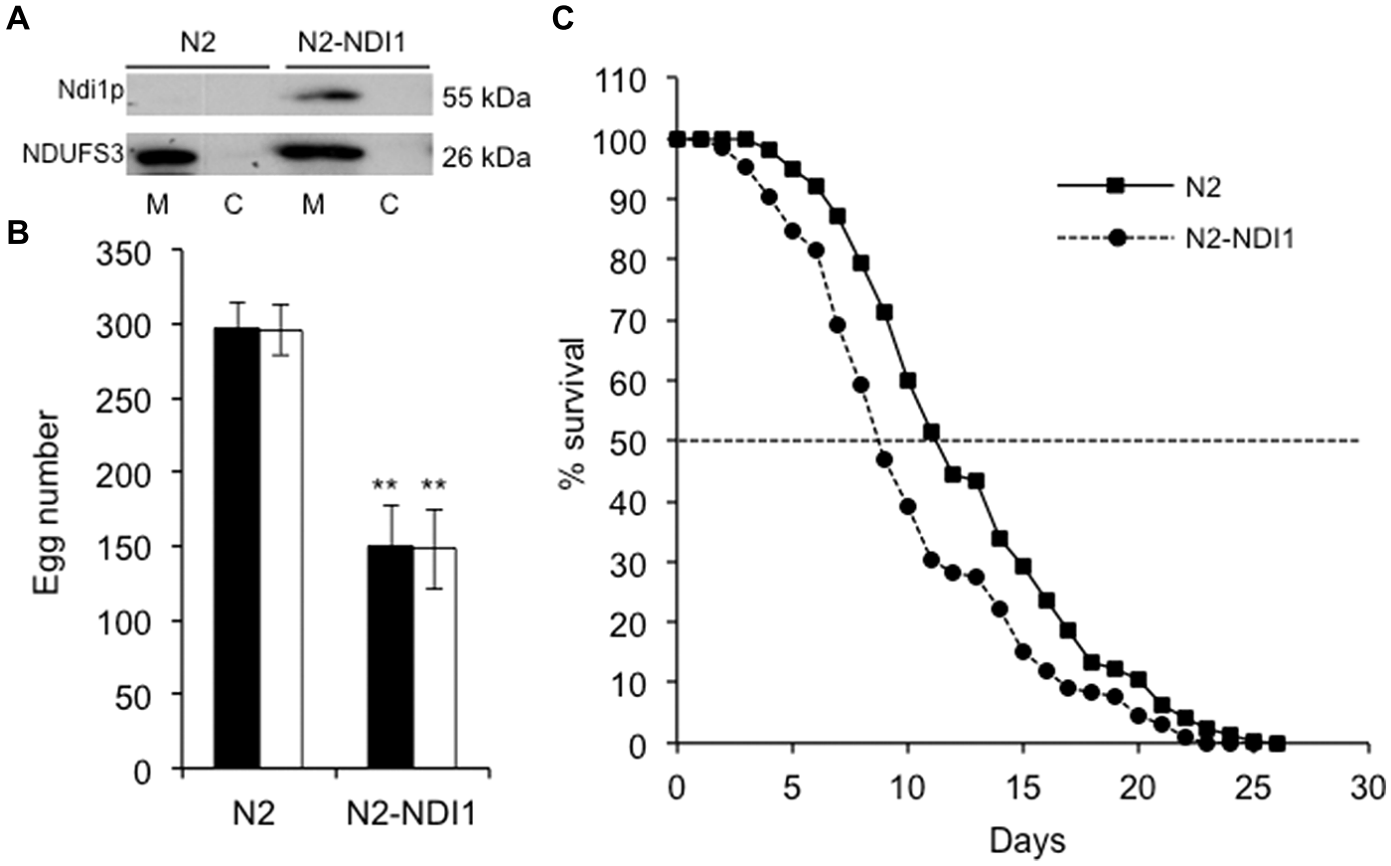
**N2-NDI1 expressing worm phenotypes. (A)** Western blot analysis of mitochondrial (M) and cytosolic (C) protein extracts from N2 and N2-NDI1 worms probed with antibodies directed against *Saccharomyces cerevisiae* Ndi1p and NDUFS3. For mitochondrial extracts, 20 μg protein were loaded. **(B)** Brood size measurements. Synchronized hermaphrodites were incubated at 20°C and the number of laid eggs were scored. Values are the mean of three broods counted and are representative of one of each three independent experiments performed. Black bars correspond to the number of laid eggs and white bars to the number of hatched eggs. Asterisks indicate statistical significance when N2-NDI1 is compared to N2 strain using *t*-test (^∗∗^*P* < 0.01). **(C)** Survival curves for N2 (black line) and N2-NDI1 (dotted line) animals. Surviving animals were monitored each day and scored as dead if they did not respond to stimulation. Plotted data are from three independent experiments with 100 worms each, giving similar results. Dotted line indicates extrapolation of median lifespan.

The mosaic transgenic nematode strain N2-NDI1 expresses the *S. cerevisiae NDI1* gene that encodes a single subunit NADH dehydrogenase already demonstrated to be able to compensate or at least mitigate complex I deficiency consequences in several organisms *in vivo* and *in vitro* (DeCorby et al., 2007; Maas et al., 2010; Marella et al., 2010; Sanz et al., 2010; Chadderton et al., 2013). N2-NDI1 animals express *NDI1* under the control of the strong and ubiquitous promoter *let-858*. As Ndi1p is a mitochondrial NADH dehydrogenase that does not exist in *C. elegans*, its expression was investigated by Western blot analysis using a specific antibody directed against *S. cerevisiae* Ndi1p. Because Ndi1p was not detectable in total N2-NDI1 protein extract (data not shown), we analyzed mitochondrial enriched extracts (**Figure 1A**). The detection of a signal migrating around 55 kDa in N2-NDI1, absent in wild type (N2) confirmed the proper expression and addressing of Ndi1p to mitochondria. The signal obtained with the human NDUFS3 antibody revealed that no modification in the steady state level of this complex I subunit was detected upon *NDI1* expression.

As growth, development, reproduction capability, and aging are highly dependent on a functional respiratory chain in worms (Dillin et al., 2002; Lee et al., 2003; Tsang and Lemire, 2003; Grad and Lemire, 2004; Dancy et al., 2014), these parameters were investigated. First, N2-NDI1 worms showed a decreased reproductive capability as measured by the number of laid eggs, which was reduced at least by 50%, as compared to N2 animals (**Figure 1B**, black bars). The amount of hatched eggs was scored in the same experiments (**Figure 1B**, white bars). All the eggs laid by N2 and N2-NDI1 animals hatched thus pointing out the absence of embryonic lethality in both strains and indicating that *NDI1* expression did not impaired embryogenesis.

Second, N2-NDI1 animals displayed a significant reduced median lifespan (**Figure 1C**) equal to 8.6 compared to 11 days of N2 animals (log-rank test, *P* < 0.001). Since complex I deficiency is also associated with a developmental delay (Grad and Lemire, 2004), we measured larval developmental time from L1–L4 stages. No difference was found between the N2 and N2-NDI1 worms, both displaying a larval stage development of 46 h at 20°C (data not shown).

Altogether, these results show that ectopic expression of Ndi1p in wild-type worms is not benign.

### Ndi1p Protects Nematode Against Rotenone Toxicity and Increases Rotenone-Insensitive Respiration

Rotenone (a potent specific complex I inhibitor) exposure has been described to alter significantly the survival of N2 animals (Ved et al., 2005). In opposition to complex I, Ndi1p catalyzes oxidation of mitochondrial matrix NADH in a rotenone insensitive manner (Marres et al., 1991).

**FIGURE 2:**
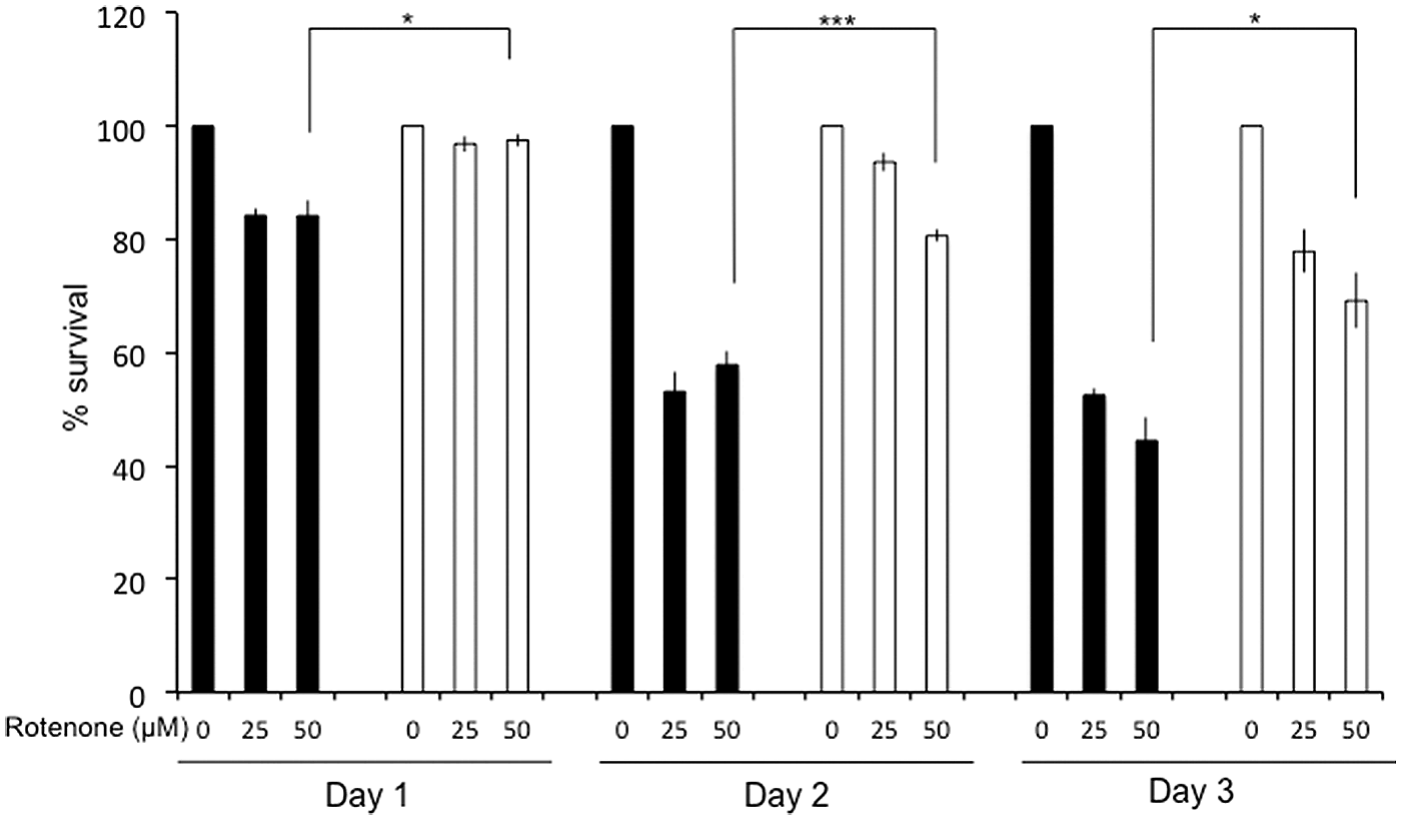
**Ndi1p protects animals against rotenone.** Fifteen N2 (black bars) and N2-NDI1 (white bars) young adult worms were exposed to 25 and 50 μM of rotenone on nematode growth medium (NGM) plates and incubated at 20°C over 3 days. Animal survival was scored each 24 h. Each histogram represents the mean values ± SEM of three independent experiments. Statistical significance was tested by *t*-test (^∗^*P* < 0.05, ^∗∗∗^*P* < 0.001) when N2-NDI1 strain is compared to N2 treated in the same conditions.

We hypothesized that *NDI1* expression should protect worms against rotenone toxicity. To test this hypothesis, N2 and N2-NDI1 synchronized young adults were exposed to 25 and 50 μM of rotenone, concentrations known to alter significantly and in a dose-dependent manner the survival of N2 animals (Ved et al., 2005). At day 1, synchronized adults were placed onto NGM plates supplemented with rotenone and worm survival was scored each day during 3 days (**Figure 2**). The two strains displayed a dose- and time-dependent reduction of viability upon rotenone treatment, but in a lesser extent in the N2-NDI1 worms. After 1-day of rotenone exposure at 25 and 50 μM, 85% of the N2 worms *versus* more than 95% of the N2-NDI1 were alive. After 48 h rotenone exposure N2 worms showed only 55% survival, whereas 80–95% of the N2-NDI1 animals were still alive. Eventually, a 72 h rotenone exposure to the same concentrations led to 48–56% mortality for N2 animals and less than 30% mortality for N2-NDI1 worms. Overall, the Ndi1p expressing nematodes showed a significantly reduced vulnerability to rotenone toxicity after 3 days of poison exposure with a higher survival percentage observed in all tested conditions giving evidence on the Ndi1p functionality.

To address the participation of Ndi1p to the worm respiration, we measured the whole live animal respiration rate, its sensitivity to rotenone, and whole animal ATP content. Respiration of N2 worms was found 60% resistant to rotenone whereas respiration of N2-NDI1 adult animals was at least 85% resistant to rotenone (**Table 1**). N2 and N2-NDI1 whole animal respiration rates and ATP content were found to be similar (**Table 1**) indicating that Ndi1p preserved the overall animal respiration and ATP production.

**Table 1:** Ndi1p does not alter whole animal respiration rate and ATP content but increases rotenone resistance.

Strain	Respiration rate^a^ (-rotenone)	Respiration rate^a^ (+rotenone)	Rotenone resistance (%)	ATP content^b^
N2	0.93 ± 0.20	0.56 ± 0.2^∗∗a^	60	8.6 ± 0.6
N2-NDI1	0.94 ± 0.15	0.80 ± 0.2	85	8.5 ± 0.5

^a^Respiration rates are expressed as nmol O_2_ consumed/min/1000 adult animals and are the means ± SD of at least six independent experiments. For rotenone sensitivity, adult animals were used and 400 μM rotenone was added into the oxygraph chamber during the rate measurement. ^∗∗a^*P* < 0.01 when compared to the non-treated parental line using a *t*-test. ^b^Whole animal ATP content was measured in L4 animals using a luciferase-based assay and is expressed as nmol/mg protein.

### Ndi1p Confers Hypersensitivity to Ethidium Bromide

**FIGURE 3:**
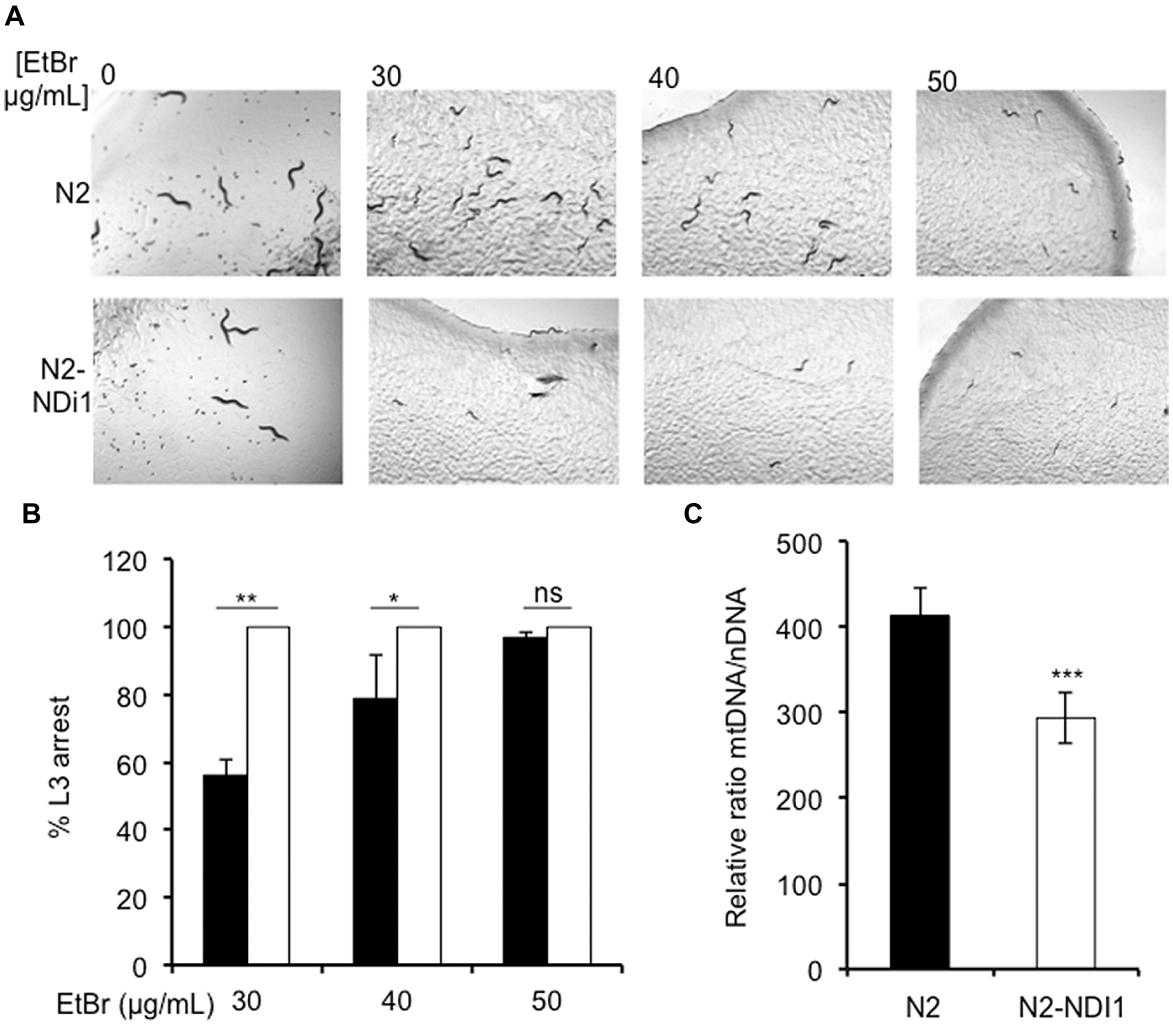
**Ndi1p expression leads to ethidium bromide (EtBr) sensitivity. (A)** L3 larval development arrest of wild type N2 (black bars) and N2-ND1 (white bars) worms after 30, 40, and 50 μg/mL EtBr exposure on NGM plates. Pictures were taken after 4 days exposure. **(B)** Percentage of L3 arrested worms after 30, 40, and 50 μg/mL of EtBr exposure. For each experiment around 100 animals N2 (black bars) and N2-ND1 (white bars) were used. Values are the mean (± SD) of three independent experiments. **(C)** mtDNA content of 9 days old adult worms in N2 (white bars) and N2-NDI1 (black bars) animals. Real time qPCR experiments were performed three times independently and the mtDNA content is expressed using *act-3* as the nuclear reference gene (means ± SEM). Asterisks indicate statistical significance in comparison to N2 (*t*-test, ^∗∗∗^*P* < 0.001).

Since in humans, several complex I subunits (NDUFS1, NDUFA9, and NDUFV3) are known to participate to the mitochondrial nucleoid, the mitochondrial DNA (mtDNA) packaging structure (Bogenhagen et al., 2008), we hypothesized that expression of Ndi1p could modify this participation generating mtDNA maintenance defects. We have previously shown that worm exposure to EtBr exacerbates the consequences of mtDNA anomalies in *C. elegans* (Addo et al., 2010). Synchronized N2 and N2-NDI1 worms were allowed to lay eggs on medium supplemented by 30, 40, and 50 μg/mL EtBr and the F1 larval development was analyzed until day 4 of adulthood (**Figures 3A,B**). As previously described, N2 worms were sensitive to EtBr exposure in a dose dependent manner and 100% of the larvae were arrested at the L3 stage in presence of 50 μg/mL EtBr. In contrast, N2-NDI1 animals were hypersensitive toward EtBr since 100% of the F1 progeny was arrested at the L3 stage from the lowest EtBr concentration tested (30 μg/mL). These results suggested that N2-NDI1 animals displayed anomalies in mtDNA content. To confirm that notion, we further quantified the relative mtDNA copy number of N2 and N2-NDI1 animals (not treated with EtBr) by real time qPCR. The amount of mtDNA in the N2-NDI1 animals was found reduced by 30% as compared to N2 worms (**Figure 3C**). Nevertheless *NDI1* expression affects mtDNA content with no impact on the N2-NDI1 worm respiration rate (**Table 1**).

### Ndi1p Alleviates the Consequences of Two Complex I Subunits Gene Silencing

*NDI1* expression is known to partially suppress some of the defects due to a point mutation in the *nuo-1* gene, the worm NDUFV1 ortholog (DeCorby et al., 2007). Here, we determined the capacity of Ndi1p to bypass RNAi induced complex I deficiencies upon inactivation of *nuo-1* and C34B2.8, the worm orthologs of human NDUFV1 and GRIM-19 (NDUFA13), respectively. Both are very well known structural genes involved in the function and the assembly of complex I (Falk et al., 2009). *nuo-1* inactivation leads to severe complex I deficiency and the mimic of human mutations in this gene was demonstrated to be pathogenic in the worm, leading to severe phenotypes (Tsang et al., 2001; Grad and Lemire, 2004). The second gene, GRIM-19 is an accessory subunit whose inactivation leads to complex I assembly defect in a mouse model (Huang et al., 2004). In *C. elegans*, a large-scale RNAi approach showed that knockdown of each of these genes causes 50 to 80% embryonic lethality of the F1 progeny when performed at the L3–L4 stage (Kamath et al., 2003).

Therefore, we applied RNAi against these two known complex I subunits either at the L1 or the L3 stage looking for the most appropriate criterion in the perspective of a large-scale RNAi screening.

We thus scored egg laying of the F0 generation for animals submitted to RNAi from the L1 stage (**Table 2**) and embryonic lethality of the F1 progeny for animals RNAi treated since the L3 stage (**Table 3**). Indeed upon RNAi treatment at the L1 stage, the significant decrease in egg laid by N2 animals makes it difficult to properly measure any embryonic lethality. N2 worms laid only 24% (NDUFV1 RNAi) and 20% (GRIM-19 RNAi) eggs as compared to the control (L4440). In contrast, N2-NDI1 animals, respectively, laid 62 and 60% eggs as compared to the not RNAi treated parental line, without any egg laying delay observed for both strains.

**Table 2:** Ndi1p compensates sterility associated with complex I deficiency.

L1 RNAi	Egg laying^a^ (%)		Respiration rates^b^	
	N2	N2-NDI1	N2	N2-NDI1
L4440	100	100	4.3 ± 0.2	4.1 ± 0.1
NDUFV1	24 ± 6	62 ± 8^∗∗∗^	2.5 ± 0.2^∗∗∗∗^	4.3 ± 0.2
GRIM-19	20 ± 1	60 ± 5^∗∗∗^	nt	nt

^a^Egg laying is given as the percentage of laid eggs by worms upon RNAi on laid eggs by the corresponding animals fed by HT115 bacteria carrying L4440 control plasmid. Values are the means (± SD) of at least two independent experiments. ^b^Values are expressed as nmol O_2_ consumed/min/1000 L4 animals and are the means ± SD of at least three independent experiments. ^∗∗∗^*P* < 0.001, ^∗∗∗∗^*P* < 0.0001 when compared to the not RNAi treated lines using a *t*-test. nt, not tested.

**Table 3:** Ndi1p compensates embryonic lethality associated with complex I deficiency.

L3 RNAi	Embryonic lethality (%)		NDUFV1 relative mRNA level		GRIM-19 relative mRNA level	
	N2	N2-NDI1	N2	N2-NDI1	N2	N2-NDI1
L4440	0	7 ± 6	1	1	1	1
NDUFV1	83 ± 14	37 ± 8^∗∗∗^	0.20 ± 0.05	0.23 ± 0.05	nt	nt
GRIM-19	27 ± 19	3 ± 3^∗^	nt	nt	0.018 ± 0.003	0.035 ± 0.010

Embryonic lethality corresponds to the percentage of eggs laid that did not hatched. Values are the means (± SD) obtain from at least three independent RNAi experiments; more than 100 eggs were scored for each experiment. A *t*-test was performed with the data obtained without RNAi treatment (*P < 0.05, ***P < 0.001). Quantification of NDUFV1 (C09H10.3) and GRIM-19 (C34B2.8) transcripts in N2 and N2-NDI grown in RNAi conditions was performed by qRT-PCR relative to *ama-1* (F36A4.7). The data are presented given an arbitrary value of 1 to the relative abundance of transcripts present in the strains submitted to control RNAi (L4440). Nt, not tested.

For RNAi applied at the L3 stage against the same subunits encoding genes, embryonic lethality level of the F1 progeny was quantified by counting at least 100 eggs laid and hatching was scored 24 h later. NDUFV1 knockdown led to 83% embryonic lethality in N2 worms while only to 37% in N2-NDI1 animals. GRIM-19 inactivation led to 27% embryonic lethality in N2 worms but it only reached 3% in N2-NDI1 animals (**Table 3**). Moreover, the F1 progeny of N2 worms submitted to RNAi against GRIM-19 from the L3 stage was blocked at the L2/L3 larval stage whereas the N2-NDI1 progeny was able to develop properly until adulthood (data not shown).

We also measured respiration rates of N2 and N2-NDI1 synchronized L4 animals subjected or not to NDUFV1 RNAi. L1 RNAi led to a 42% decreased of the N2 respiration rate whereas no decrease was observed for N2-NDI1 animals subjected to the same RNAi (**Table 2**). This result suggested that Ndi1p is able to restore the respiration rate decrease due to complex I deficiency and thus to compensate the complex I deficiency associated phenotype. NDUFV1 and GRIM-19 knock down efficiencies were checked at the mRNA level by real time RT-qPCR in N2 and N2-NDI1 animals (**Table 3**). A similar reduction of mRNA level was found between the two lines indicating that the beneficial effect observed in the N2-NDI1 animals was not due to a less efficient RNAi in this line.

As we selected N2-NDI1 worms for their G418 resistance rather than sorting them thanks to their GFP staining for technical convenience, we evaluated the innocuousness of G418 treatment on RNAi efficiency. We applied L3 RNAi protocol on N2-NDI1 larvae selected either by G418 exposure or GFP staining and scored embryonic lethality on the F1 progeny. We found a comparable beneficial Ndi1p effect in the two groups (Supplementary Figure S1).

Altogether, these results establish the beneficial effect of Ndi1p expression on egg-laying default, embryonic lethality, and larval developmental arrest due to the RNAi knockdown of two complex I subunit encoding genes, applied either at the L1 or L3 stage.

### Ndi1p Complements the Embryonic Lethality Upon RNAi Targeting Several Genes Encoding Complex I Catalytic and Accessory Subunits

**FIGURE 4:**
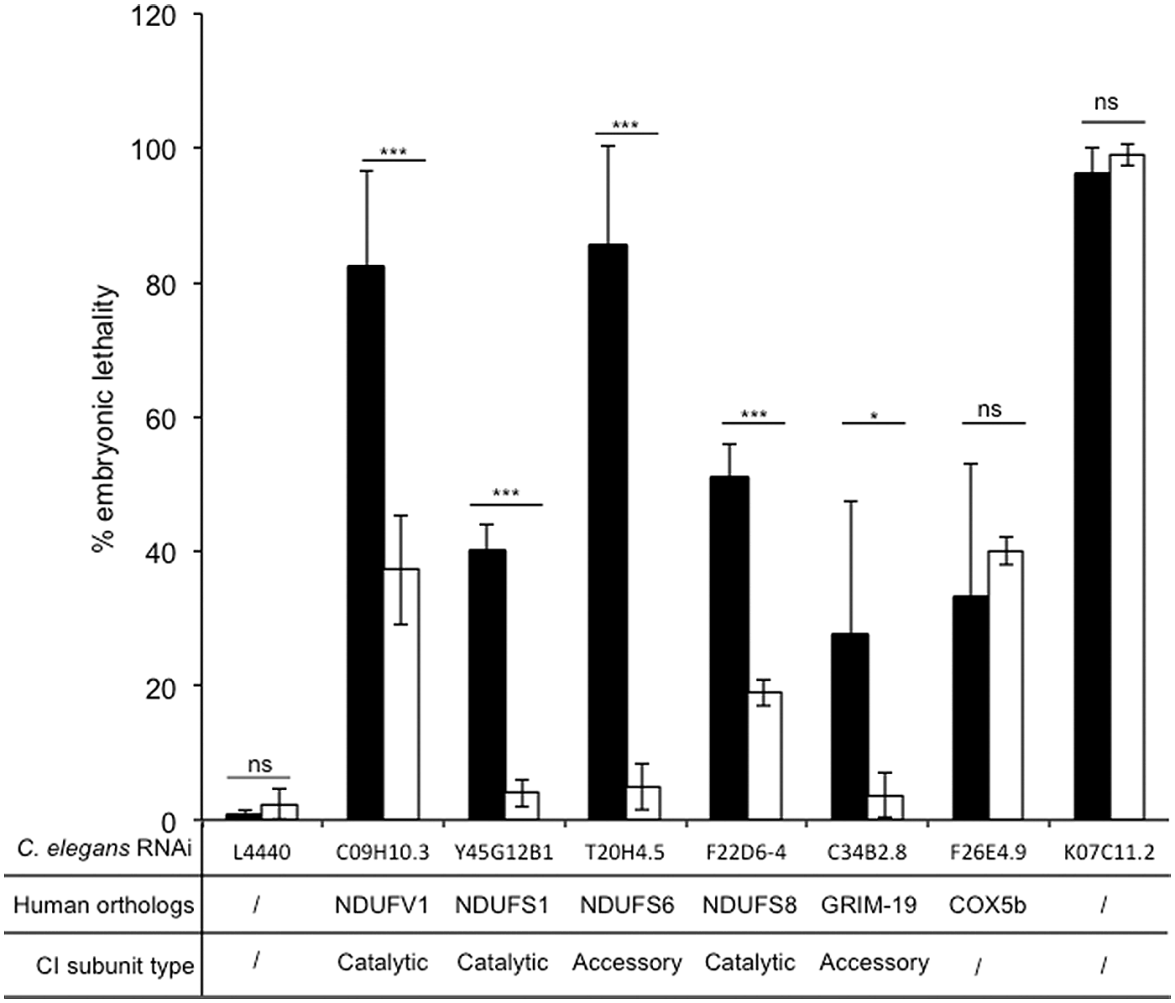
**Ndi1p decreases specifically embryonic lethality due to RNAi knockdown of complex I subunits.** Embryonic lethality was calculated, as the percentage of eggs laid that did not hatched. Black bars (N2) and white bars (N2-NDI1) represent the means (± SD) of embryonic lethality of at least three independent experiments with more than 100 eggs laid scored by experiment. RNAi knockdown targets were C09H10.3, C34B2.8, Y45G12B1, T20H4.5, F22D6-4, F26E4.9, and K07C11.2. Asterisks show statistical significance (*t*-test, *P < 0.05, ***P < 0.001) when N2-NDI1 was compared with N2 treated by identical RNAi.

We further inactivated the expression of several genes encoding known catalytic and accessory complex I subunits to confirm complementation property of Ndi1p. We used the L3 RNAi protocol validated previously. Among known genes, we selected those that lead either to mild (<50%) or to strong (>50%) embryonic lethality upon RNAi inactivation in a large-scale screening, in order to determine to which extent Ndi1p would be able to save embryonic lethality. In addition to NDUFV1 and GRIM-19 we decided to inactivate expression of three additional complex I related genes Y45G12B1, T20H4.5, and F22D6-4 which are the orthologs of human NDUFS1, NDUFS6, and NDUFS8 genes, respectively. All these genes encode subunit involved in complex I structure, with different functions: (i) NDUFV1 and NDUFS1 are catalytic subunits involved in the N module, (ii) NDUFS8 is also a catalytic subunit but involved in the Q module, (iii) NDUFS6 and GRIM-19 are accessory subunits. In human, a homozygous NDUFS6 mutation leads to a decreased amount of fully assembled complex I and accumulation of a subcomplex (Kirby et al., 2004), while in mice, GRIM-19 knockdown neither assembled complex I nor accumulated subcomplex is observed (Huang et al., 2004; Pagniez-Mammeri et al., 2012a). In parallel, we checked the specificity of the Ndi1p bypass by treating worms with RNAi directed against two non-related complex I genes. F26E4.9, which encodes the human cytochrome *c* oxidase subunit 5b ortholog (complex IV subunit; Tsang and Lemire, 2003) and K07C11.2, a non mitochondrial gene encoding a serine-threonine kinase involved in the assembly of the mitotic spindle microtubule (Kamath et al., 2003; Toya et al., 2011). As shown in **Figure 4**, all N2 worms fed by HT115 bacteria allowing RNAi were characterized by embryonic lethality in the expected range of 30 to 95%. For N2 worms, NDUFV1, NDUFS6, and K07C11.2 inactivation led to more than 80% embryonic lethality and NDUFS1, NDUFS8, GRIM-19, and COX5b inactivation led to less than 50% embryonic lethality. In N2-NDI1 worms the level of embryonic lethality was significantly reduced whatever the complex I related gene silenced and the embryonic lethality due to the non-complex I related genes silencing was not diminished underlying the complex I specificity of the Ndi1p bypass.

## Discussion

In this paper we propose a new, rapid, and cheap strategy to find out new genes involved in complex I function taking advantage of a *C. elegans* worm expressing the *S. cerevisiae NDI1* gene, that encodes a single subunit NADH dehydrogenase able to bypass complex I. We first characterized the N2-NDI1 nematode strain (expressing Ndi1p). Then we checked the Ndi1p capacity to specifically overcome consequences of known complex I subunits knocked down by RNAi in order to prove the feasibility of an incoming large-scale screening.

N2-NDI1 animals are characterized by a preserved whole animal respiration rate, ATP content, and developmental schedule while Ndi1p appears to be properly expressed and targeted to mitochondria as assessed by Western blot analysis. Furthermore, Ndi1p expression confers an increased resistance to rotenone suggesting the functionality of Ndi1p at least in a complex I deficient context.

Nonetheless, N2-NDI1 animals present a decreased progeny number by 50%, a significantly reduced lifespan and a subtle 30% mtDNA content decrease. However, the electron transport chain content seems to be preserved as NDUFS3 steady-state level and whole animal respiration rate are similar in N2 and N2-NDI1 animals. Thus, in presence of a functional complex I, expression of Ndi1p is somehow deleterious. On the contrary, when complex I is impaired by the silencing of one of its subunits or by the use of rotenone, expression of Ndi1p has beneficial effects. This indicates that Ndi1p is functional and may compete with complex I.

Because complex I is absent in the yeast *S. cerevisiae*, it might be predicted that Ndi1p has similar kinetic properties as complex I toward NADH. Ndi1p is an enzyme that does not translocate protons, therefore the fraction of electrons transiting through Ndi1p will not participate to ATP production. Consequently, the respiration rate and ATP production should be impacted. In order to explain the phenotypic differences between N2 and N2-NDI1 animals despite similar ATP content (at the whole animal level), we can hypothesize that in presence of a functional complex I, the impact of Ndi1p on respiration rate and ATP production is low and not detectable at the level of the whole mosaic animals. However, in *NDI1*-expressing cells, such as the germline, deleterious consequences on egg production might be expected if ATP decreases under a threshold level (reduced electron flow through complex I) or if reactive oxygen species are overproduced (increased global electron flux). Interestingly, oxidative stress associated with a reduced brood size and a slightly reduced lifespan have been described in *nuo-1* mutant worms (Grad and Lemire, 2004), phenotypes also exhibited by N2-NDI1 animals.

Altogether, we showed that the expression of the *S. cerevisiae* internal NADH dehydrogenase Ndi1p, in a wild type nematode is not benign. On the contrary, Ndi1p expression is described to be largely innocuous in *Drosophila* conferring only a slightly increased lifespan (Sanz et al., 2010). In the N2-NDI1 worm, the transgene *NDI1* is under the control of a strong ubiquitous promoter (*let-858*) and carried on an extrachromosomal array. This latter is a high molecular weight DNA concatemer composed of up to hundreds of copies of the plasmid harboring the transgene. It might lead to Ndi1p overexpression that can be toxic (Stinchcomb et al., 1985), but may be attenuated by the mosaicity of the animal. In *Drosophila*, the *NDI1* transgene is under the control of an inducible promoter and only 1–4 copies are inserted into the genome. Consequently, Ndi1p amount in N2-NDI1 nematode might be much higher and present during all the worm life compared to *Drosophila*; these differences could therefore explain the toxicity reported in the worm.

It would be of interest to construct a new N2-NDI1 nematode in which the transgene expression level can be controlled or a new N2-NDI1 worm expressing an alternative NADH dehydrogenase which naturally co-exists with a complex I, as in plants and many fungi.

We also showed that Ndi1p expressed in wild type animals rescued phenotypes caused by several complex I subunit RNAi knockdowns, confirming the pioneer works published by Lemire and co-workers (DeCorby et al., 2007). RNAi of C09H10.3 (NDUFV1) and C34B2.8 (GRIM-19) in N2 worms, applied from the L1 or the L3 stage, led, respectively, to severe egg laying defect or embryonic lethality. In N2-NDI1 animals submitted to the same RNAi conditions, these phenotypes were both impressively alleviated. Moreover, the L3 stage arrest observed in the F1 progeny of N2 worms treated by RNAi against GRIM-19 from the L3 stage was entirely complemented by Ndi1p indicating the beneficial effect of his protein on complex I deficiency whatever the phenotype was.

We further demonstrated the Ndi1p complementation capability over structural or accessory complex I subunits belongings to the functional domains involved in NADH oxidation (N module) and ubiquinone reduction (Q module; ortologs of NDUFS1, NDUFS6, and NDUFS8). These results make us confident in the efficiency of the screening we propose to implement by the use of embryonic lethal phenotype based sub-genomic library derived from the genomic RNAi library (Kamath et al., 2003). If we are conscious that we will miss assembly factors obviously absent from the embryonic lethal sub-library as the worms orthologs of human NDUFAF1, NDUFAF3 genes (van den Ecker et al., 2012) and that we will probably not identify new additional catalytic subunits, we remain quite confident that we will identify many other complex I related genes such as: (i) key assembly factors whose knockdown could lead to drastic complex I activity decrease; (ii) new accessory subunit loosely associated to complex I but important for complex I activity; (iii) modulators of complex I that can act transcriptionally or post-translationally such as STAT3 and PINK1 identified in human. STAT3 is a transcription factor demonstrated to interact with complex I and to negatively modulate complex I activity (Wegrzyn et al., 2009). PINK1 is a mitochondrial serine/threonine kinase involved directly or not in the phosphorylation of NDUFA10, known to be crucial for the regulation of the ubiquinone reduction (Morais et al., 2014). STAT3 and PINK1 are probably not the only proteins able to regulate complex I activity and the screening we proposed should reveal such complex I activity modulators depending on enzymatic modifications such as phosphorylation and acetylation. Interestingly, STAT transcription factors and PINK1 are conserved in *C. elegans* underlying the possibility to identify that category of genes in our model (Wang and Levy, 2006; Samann et al., 2009).

**FIGURE 5:**
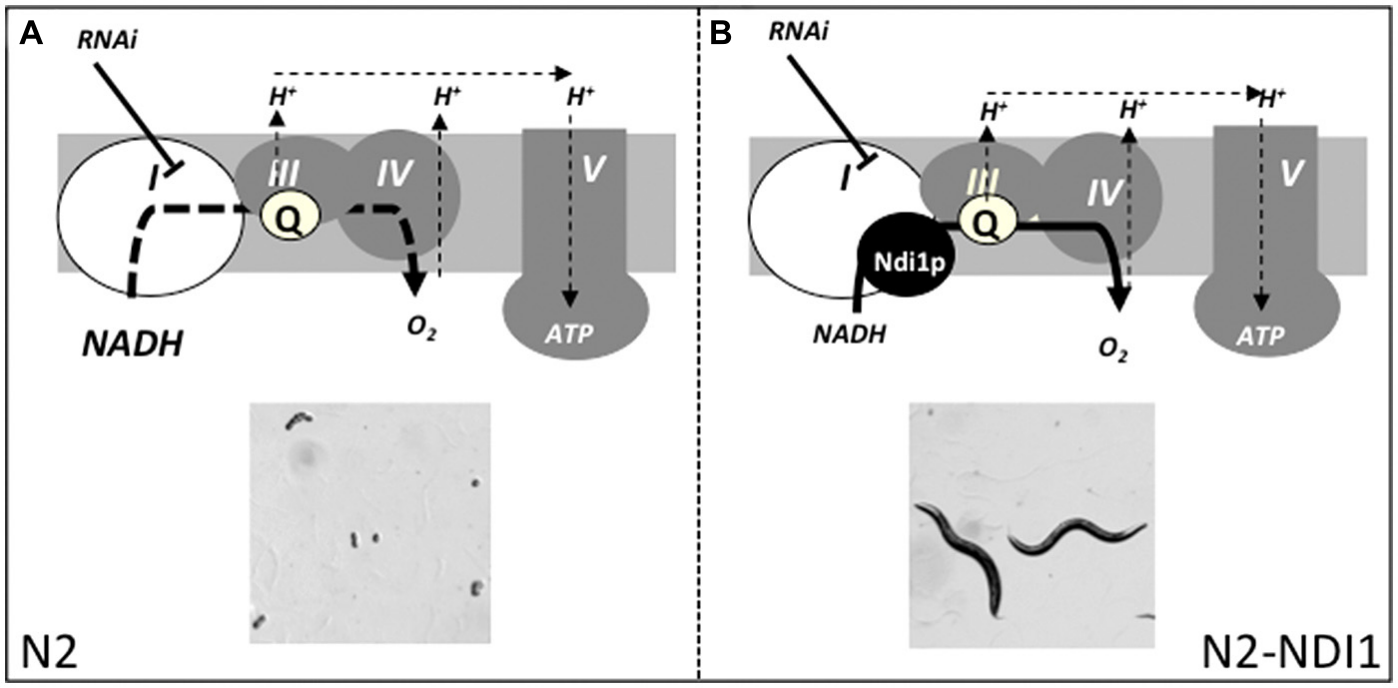
**RNAi-based complex I subunits screen strategy.** N2 and N2-NDI1 animals will be subjected to RNAi from an embryonic lethal sub-genomic library. We hypothesized that complex I gene inactivation will lead to embryonic lethality in N2 reference strain **(A)** and not or in a lesser extent in N2-NDI1 animals allowing eggs to hatch and animals to develop **(B)**. I–V figure the canonical respiratory chain complexes; Q, ubiquinone; bold dotted and plain line represent altered or active electron flow and thin dotted lines represents proton translocation. Adapted from Rustin and Jacobs (2009).

Eventually, given: (i) that embryonic lethality is the most often observed phenotype in RNAi induced complex I deficient worms, (ii) the existence of an embryonic lethal sub-genomic RNAi library, and (iii) the Ndi1p complementation ability on embryonic lethality due to RNAi of five different known complex I genes, we decided to apply the L3 RNAi approach scoring the F1 embryonic lethality progeny to identify new complex I related genes in N2 and N2-NDI1 worms. We hypothesized that the embryonic lethality in N2 worms would be at least alleviated in N2-NDI1 animals, thanks to the shortcut offered by Ndi1p, pointing to new complex I related genes (**Figure 5**).

By the use of such a strategy, we hope to identify new genes involved in complex I activity, regulation and assembly that would be candidate genes to validate in human patients presenting a complex I deficiency without known corresponding molecular basis. The identification of these new genes would also help to get a better understanding of complex I.

## Conflict of Interest Statement

The authors declare that the research was conducted in the absence of any commercial or financial relationships that could be construed as a potential conflict of interest.

## References

[B1] Addo M. G. Cossard R. Pichard D. Obiri-Danso K. Rotig A. Delahodde A. (2010). *Caenorhabditis elegans*, a pluricellular model organism to screen new genes involved in mitochondrial genome maintenance. *Biochim. Biophys. Acta* 1802 765–773. 10.1016/j.bbadis.2010.05.007 20580819

[B2] Bogenhagen D. F. Rousseau D. Burke S. (2008). The layered structure of human mitochondrial DNA nucleoids. *J. Biol. Chem.* 283 3665–3675. 10.1074/jbc.M708444200 18063578

[B3] Braeckman B. P. Houthoofd K. De Vreese A. Vanfleteren J. R. (2002). Assaying metabolic activity in ageing *Caenorhabditis elegans*. *Mech. Ageing Dev.* 123 105–119. 10.1016/S0047-6374(01)00331-1 11718805

[B4] Brandt U. (2006). Energy converting NADH:quinone oxidoreductase (complex I). *Annu. Rev. Biochem.* 75 69–92. 10.1146/annurev.biochem.75.103004.142539 16756485

[B5] Cannino G. El-Khoury R. Pirinen M. Hutz B. Rustin P. Jacobs H. T. (2012). Glucose modulates respiratory complex I activity in response to acute mitochondrial dysfunction. *J. Biol. Chem.* 287 38729–38740. 10.1074/jbc.M112.386060M112.386060 23007390 PMC3493916

[B6] Chadderton N. Palfi A. Millington-Ward S. Gobbo O. Overlack N. Carrigan M. (2013). Intravitreal delivery of AAV-NDI1 provides functional benefit in a murine model of Leber hereditary optic neuropathy. *Eur. J. Hum. Genet.* 21 62–68. 10.1038/ejhg.2012.112 22669418 PMC3522193

[B7] Cho J. Hur J. H. Graniel J. Benzer S. Walker D. W. (2012). Expression of yeast NDI1 rescues a *Drosophila* complex I assembly defect. *PLoS ONE* 7:e50644 10.1371/journal.pone.0050644 PMC3511326 23226344

[B8] Dancy B. M. Sedensky M. M. Morgan P. G. (2014). Effects of the mitochondrial respiratory chain on longevity in *C. elegans*. *Exp. Gerontol.* 56 245–255. 10.1016/j.exger.2014.03.028 24709342

[B9] DeCorby A. Gaskova D. Sayles L. C. Lemire B. D. (2007). Expression of Ndi1p, an alternative NADH:ubiquinone oxidoreductase, increases mitochondrial membrane potential in a *C. elegans* model of mitochondrial disease. *Biochim. Biophys. Acta* 1767 1157–1163. 10.1016/j.bbabio.2007.07.003 17706937

[B10] Dillin A. Hsu A. L. Arantes-Oliveira N. Lehrer-Graiwer J. Hsin H. Fraser A. G. (2002). Rates of behavior and aging specified by mitochondrial function during development. *Science* 298 2398–2401. 10.1126/science.10777801077780 12471266

[B11] Falk M. J. Rosenjack J. R. Polyak E. Suthammarak W. Chen Z. Morgan P. G. (2009). Subcomplex Ilambda specifically controls integrated mitochondrial functions in *Caenorhabditis elegans*. *PLoS ONE* 4:e6607 10.1371/journal.pone.0006607 PMC2719872 19672299

[B12] Fassone E. Rahman S. (2012). Complex I deficiency: clinical features, biochemistry and molecular genetics. *J. Med. Genet.* 49 578–590. 10.1136/jmedgenet-2012-101159 22972949

[B13] Grad L. I. Lemire B. D. (2004). Mitochondrial complex I mutations in *Caenorhabditis elegans* produce cytochrome c oxidase deficiency, oxidative stress and vitamin-responsive lactic acidosis. *Hum. Mol. Genet.* 13 303–314. 10.1093/hmg/ddh027 14662656

[B14] Haack T. B. Madignier F. Herzer M. Lamantea E. Danhauser K. Invernizzi F. (2012). Mutation screening of 75 candidate genes in 152 complex I deficiency cases identifies pathogenic variants in 16 genes including NDUFB9. *J. Med. Genet.* 49 83–89. 10.1136/jmedgenet-2011-100577 22200994

[B15] Hoefs S. J. Rodenburg R. J. Smeitink J. A. Van Den Heuvel L. P. (2012). Molecular base of biochemical complex I deficiency. *Mitochondrion* 12 520–532. 10.1016/j.mito.2012.07.106 22820119

[B16] Hoogewijs D. Houthoofd K. Matthijssens F. Vandesompele J. Vanfleteren J. R. (2008). Selection and validation of a set of reliable reference genes for quantitative sod gene expression analysis in *C. elegans*. *BMC Mol. Biol.* 9:9 10.1186/1471-2199-9-9 PMC2254638 18211699

[B17] Huang G. Lu H. Hao A. Ng D. C. Ponniah S. Guo K. (2004). GRIM-19 a cell death regulatory protein, is essential for assembly and function of mitochondrial complex I. *Mol. Cell. Biol.* 24 8447–8456. 10.1128/MCB.24.19.8447-8456.2004 15367666 PMC516758

[B18] Kamath R. S. Ahringer J. (2003). Genome-wide RNAi screening in *Caenorhabditis elegans*. *Methods* 30 313–321. 10.1016/S1046-2023(03)00050-1 12828945

[B19] Kamath R. S. Fraser A. G. Dong Y. Poulin G. Durbin R. Gotta M. (2003). Systematic functional analysis of the *Caenorhabditis elegans* genome using RNAi. *Nature* 421 231–237. 10.1038/nature01278 12529635

[B20] Kirby D. M. Salemi R. Sugiana C. Ohtake A. Parry L. Bell K. M. (2004). NDUFS6 mutations are a novel cause of lethal neonatal mitochondrial complex I deficiency. *J. Clin. Invest.* 114 837–845. 10.1172/JCI20683 15372108 PMC516258

[B21] Lai C. H. Chou C. Y. Ch’ang L. Y. Liu C. S. Lin W. (2000). Identification of novel human genes evolutionarily conserved in *Caenorhabditis elegans* by comparative proteomics. *Genome Res.* 10 703–713. 10.1101/gr.10.5.703 10810093 PMC310876

[B22] Lee S. S. Lee R. Y. Fraser A. G. Kamath R. S. Ahringer J. Ruvkun G. (2003). A systematic RNAi screen identifies a critical role for mitochondria in *C. elegans* longevity. *Nat. Genet.* 33 40–48. 10.1038/ng1056 12447374

[B23] Luttik M. A. Overkamp K. M. Kotter P. De Vries S. Van Dijken J. P. Pronk J. T. (1998). The *Saccharomyces cerevisiae* NDE1 and NDE2 genes encode separate mitochondrial NADH dehydrogenases catalyzing the oxidation of cytosolic NADH. *J. Biol. Chem.* 273 24529–24534. 10.1074/jbc.273.38.24529 9733747

[B24] Maas M. F. Sellem C. H. Krause F. Dencher N. A. Sainsard-Chanet A. (2010). Molecular gene therapy: overexpression of the alternative NADH dehydrogenase NDI1 restores overall physiology in a fungal model of respiratory complex I deficiency. *J. Mol. Biol.* 399 31–40. 10.1016/j.jmb.2010.04.015 20398675

[B25] Marella M. Seo B. B. Nakamaru-Ogiso E. Greenamyre J. T. Matsuno-Yagi A. Yagi T. (2008). Protection by the NDI1 gene against neurodegeneration in a rotenone rat model of Parkinson’s disease. *PLoS ONE* 3:e1433 10.1371/journal.pone.0001433 PMC2175531 18197244

[B26] Marella M. Seo B. B. Thomas B. B. Matsuno-Yagi A. Yagi T. (2010). Successful amelioration of mitochondrial optic neuropathy using the yeast NDI1 gene in a rat animal model. *PLoS ONE* 5:e11472 10.1371/journal.pone.0011472 PMC2900204 20628600

[B27] Marres C. A. De Vries S. Grivell L. A. (1991). Isolation and inactivation of the nuclear gene encoding the rotenone-insensitive internal NADH: ubiquinone oxidoreductase of mitochondria from *Saccharomyces cerevisiae*. *Eur. J. Biochem.* 195 857–862. 10.1111/j.1432-1033.1991.tb15775.x 1900238

[B28] Morais V. A. Haddad D. Craessaerts K. De Bock P. J. Swerts J. Vilain S. (2014). PINK1 loss-of-function mutations affect mitochondrial complex I activity via NdufA10 ubiquinone uncoupling. *Science* 344 203–207. 10.1126/science.1249161 24652937

[B29] Pagniez-Mammeri H. Loublier S. Legrand A. Benit P. Rustin P. Slama A. (2012a). Mitochondrial complex I deficiency of nuclear origin I. Structural genes. *Mol. Genet. Metab.* 105 163–172. 10.1016/j.ymgme.2011.11.188 22142868

[B30] Pagniez-Mammeri H. Rak M. Legrand A. Benit P. Rustin P. Slama A. (2012b). Mitochondrial complex I deficiency of nuclear origin II. Non-structural genes. *Mol. Genet. Metab.* 105 173–179. 10.1016/j.ymgme.2011.10.001 22099533

[B31] Rasmusson A. G. Geisler D. A. Moller I. M. (2008). The multiplicity of dehydrogenases in the electron transport chain of plant mitochondria. *Mitochondrion* 8 47–60. 10.1016/j.mito.2007.10.004 18033742

[B32] Rea S. L. Graham B. H. Nakamaru-Ogiso E. Kar A. Falk M. J. (2010). Bacteria, yeast, worms, and flies: exploiting simple model organisms to investigate human mitochondrial diseases. *Dev. Disabil. Res. Rev.* 16 200–218. 10.1002/ddrr.114 20818735 PMC3628736

[B33] Rotig A. (2010). Genetic bases of mitochondrial respiratory chain disorders. *Diabetes Metab.* 36 97–107. 10.1016/j.diabet.2009.11.002 20093061

[B34] Rustin P. Jacobs H. T. (2009). Respiratory chain alternative enzymes as tools to better understand and counteract respiratory chain deficiencies in human cells and animals. *Physiol. Plant.* 137 362–370. 10.1111/j.1399-3054.2009.01249.x 19508504

[B35] Saha S. Guillily M. D. Ferree A. Lanceta J. Chan D. Ghosh J. (2009). LRRK2 modulates vulnerability to mitochondrial dysfunction in *Caenorhabditis elegans*. *J. Neurosci.* 29 9210–9218. 10.1523/JNEUROSCI.2281-09.2009 19625511 PMC3127548

[B36] Samann J. Hegermann J. Von Gromoff E. Eimer S. Baumeister R. Schmidt E. (2009). Caenorhabditits elegans LRK-1 and PINK-1 act antagonistically in stress response and neurite outgrowth. *J. Biol. Chem.* 284 16482–16491. 10.1074/jbc.M808255200 19251702 PMC2713553

[B37] Sanz A. Soikkeli M. Portero-Otin M. Wilson A. Kemppainen E. Mcilroy G. (2010). Expression of the yeast NADH dehydrogenase Ndi1 in *Drosophila* confers increased lifespan independently of dietary restriction. *Proc. Natl. Acad. Sci. U.S.A.* 107 9105–9110. 10.1073/pnas.0911539107 20435911 PMC2889079

[B38] Scaglia F. Towbin J. A. Craigen W. J. Belmont J. W. Smith E. O. Neish S. R. (2004). Clinical spectrum, morbidity, and mortality in 113 pediatric patients with mitochondrial disease. *Pediatrics* 114 925–931. 10.1542/peds.2004-0718 15466086

[B39] Stinchcomb D. T. Shaw J. E. Carr S. H. Hirsh D. (1985). Extrachromosomal DNA transformation of *Caenorhabditis elegans*. *Mol. Cell. Biol.* 5 3484–3496. 3837845 10.1128/mcb.5.12.3484-3496.1985 PMC369179

[B40] Toya M. Terasawa M. Nagata K. Iida Y. Sugimoto A. (2011). A kinase-independent role for Aurora A in the assembly of mitotic spindle microtubules in *Caenorhabditis elegans* embryos. *Nat. Cell Biol.* 13 708–714. 10.1038/ncb2242ncb2242 21572421

[B41] Tsang W. Y. Lemire B. D. (2003). The role of mitochondria in the life of the nematode, *Caenorhabditis elegans*. *Biochim. Biophys. Acta* 1638 91–105. 10.1016/S0925-4439(03)00079-6 12853115

[B42] Tsang W. Y. Sayles L. C. Grad L. I. Pilgrim D. B. Lemire B. D. (2001). Mitochondrial respiratory chain deficiency in *Caenorhabditis elegans* results in developmental arrest and increased life span. *J. Biol. Chem.* 276 32240–32246. 10.1074/jbc.M103999200 11410594

[B43] van den Ecker D. Van Den Brand M. A. Ariaans G. Hoffmann M. Bossinger O. Mayatepek E. (2012). Identification and functional analysis of mitochondrial complex I assembly factor homologues in *C. elegans*. *Mitochondrion* 12 399–405. 10.1016/j.mito.2012.01.003 22387847

[B44] van den Ecker D. Van Den Brand M. A. Bossinger O. Mayatepek E. Nijtmans L. G. Distelmaier F. (2010). Blue native electrophoresis to study mitochondrial complex I in *C. elegans. Anal. Biochem.* 407 287–289. 10.1016/j.ab.2010.08.009 20705045

[B45] Ved R. Saha S. Westlund B. Perier C. Burnam L. Sluder A. (2005). Similar patterns of mitochondrial vulnerability and rescue induced by genetic modification of alpha-synuclein, parkin, and DJ-1 in *Caenorhabditis elegans*. *J. Biol. Chem.* 280 42655–42668. 10.1074/jbc.M505910200 16239214 PMC3910375

[B46] Wang Y. Levy D. E. (2006). C. elegans STAT: evolution of a regulatory switch. *FASEB J.* 20 1641–1652. 10.1096/fj.06-6051com 16873887

[B47] Wegrzyn J. Potla R. Chwae Y. J. Sepuri N. B. Zhang Q. Koeck T. (2009). Function of mitochondrial Stat3 in cellular respiration. *Science* 323 793–797. 10.1126/science.11645511164551 19131594 PMC2758306

[B48] Yagi T. Seo B. B. Nakamaru-Ogiso E. Marella M. Barber-Singh J. Yamashita T. (2006). Can a single subunit yeast NADH dehydrogenase (Ndi1) remedy diseases caused by respiratory complex I defects? *Rejuvenation Res.* 9 191–197. 10.1089/rej.2006.9.191 16706641

