# Supplementary material for: Caenorhabditis elegans expressing the Saccharomyces cerevisiae NADH alternative dehydrogenase Ndi1p, as a tool to identify new genes involved in complex I related diseases
Source: Front Genet. 2015 Jun 11;6:206. doi: 10.3389/fgene.2015.00206 (PMC4463008; doi:10.3389/fgene.2015.00206)
Supplement: Supplementary file 1 [file Image_1.PDF]

Supplementary Material

Caenorhabditis elegans expressing the *Saccharomyces cerevisiae* NADH alternative dehydrogenase Ndi1p, as a tool to identify new genes involved in complex I related diseases

Raynald Cossard[1♯], Michela Esposito[1♯], Carole H. Sellem[1], Laras Pitayu[1], Christelle Vasnier[1], Agnès Delahodde[1], Emmanuel P. Dassa[1*]

[1]Institute for Integrative Biology of the Cell (I2BC), CEA, CNRS, Université Paris-Sud, Bat 400, rue Gregor Mendel, 91405 ORSAY Cedex France
[♯]These authors contributed equally to this work.

* Correspondence
emmanuel.dassa@u-psud.fr
Dr. Emmanuel DASSA
Institute for Integrative Biology of the Cell (I2BC), CEA, CNRS, Université Paris-Sud
Rue Gregor Mendel, Bat 400, 91405 ORSAY Cedex

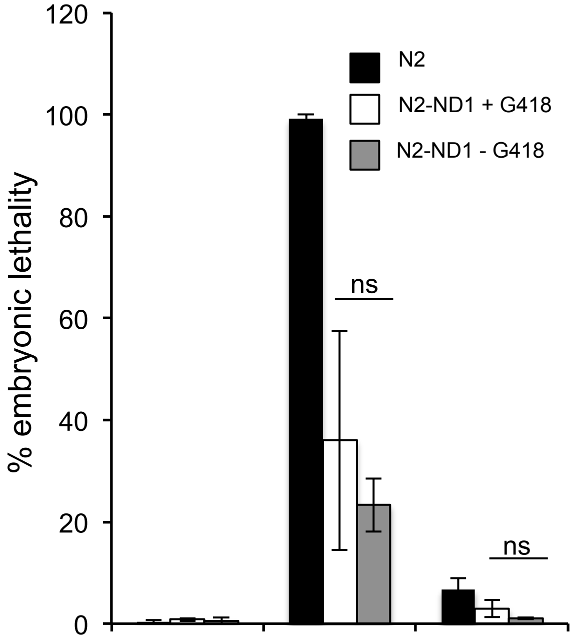

Figure S1: Effect of G418 treatment on N2-NDI rescuing capability
N2 (Black bars) and N2-NDI1 L3-L4 larvae were submitted to RNAi silencing the C09H10.3 or the C34B2.8 genes. N2-NDI worm were either previously selected between the L1-L3 stages on G418 containing NGM medium (white bars) or sorted out at the adult stage under a fluorescence stereomicroscope according to their GFP staining (grey bars). Histograms represent the means of embryonic lethality ± SD of at least three independent RNAi experiments with at least 100 eggs laid scored by experiment. Embryonic lethality was calculated as the percentage of eggs laid that did not hatched. Significance was tested by *t*-test (ns, not significant).
